# The Schizotypal Ambivalence Scale: An Item Response Theory Analysis

**DOI:** 10.3390/bs12080247

**Published:** 2022-07-22

**Authors:** Lauren B. Deters, Paul J. Silvia, Thomas R. Kwapil

**Affiliations:** 1 Workera.ai, Palo Alto, CA 94306, USA; lauren.deters@gmail.com; 2 Department of Psychology, University of North Carolina at Greensboro, Greensboro, NC 26170, USA; trkwapil@illinois.edu; 3 Department of Psychology, University of Illinois at Urbana-Champaign, Champaign, IL 61801, USA

**Keywords:** schizotypy, ambivalence, Schizotypal Ambivalence Scale, item response theory, differential item functioning

## Abstract

Ambivalence has a prominent role in the historical formulations of schizotypy and schizophrenia, as well as borderline personality disorder. However, it has been overlooked by our current diagnostic nomenclature. The Schizotypal Ambivalence Scale (SAS) is a 19-item self-report scale developed to examine ambivalence relevant to schizotypy and schizophrenia-spectrum disorders. Questionnaire, interview, and ambulatory assessment studies support the construct validity of the measure as a predictor of schizophrenia-spectrum and borderline psychopathology. However, studies have not adequately examined the item properties and factor structure of the scale. To examine the psychometric features of the SAS, the present research applied item response theory and differential item functioning methods using a large sample of adults (*n* = 7096). Analyses of dimensionality were consistent with essential unidimensionality, and a 2PL IRT model found good item discrimination, an appropriate range of item difficulty, minimal local dependence, and excellent item fit. Analyses of differential item functioning found essentially no bias for gender on any items and very small effects for two items for racial/ethnic identity. Overall, the analyses reveal many psychometric strengths of the Schizotypal Ambivalence Scale and support its use a single-factor instrument for assessing ambivalence in diverse subgroups of adults.

## 1. Introduction

Schizotypy represents the phenotypic expression of the underlying vulnerability for schizophrenia-spectrum disorders that is expressed across a broad range of clinical and subclinical impairment [1]. Schizotypy offers a useful construct for understanding the risk for and development of schizophrenia-spectrum psychopathology because it encompasses subclinical manifestations, the psychosis prodrome, schizophrenia-spectrum personality disorders, and full-blown psychosis. Schizotypy is a multidimensional construct with positive (psychotic-like), negative (deficit), and disorganized dimensions. The construct involves disruptions in cognition (e.g., magical beliefs and cognitive slippage), perception (e.g., illusions and bodily aberrations), the experience and expression of emotion (e.g., flattened or dysregulated affect), and social functioning (e.g., suspiciousness, social disinterest, and social aversiveness). 

Scholars have also suggested that *ambivalence* characterizes subclinical and clinical manifestations of schizotypy. The term ambivalence was originally coined by Bleuler [2] to represent the simultaneous experience of both positive and negative emotions and the inability to integrate these emotions. He viewed ambivalence as a manifestation of thought disorder and also considered it to be one of the core components of schizophrenia. Meehl [3] initially proposed that ambivalence was one of four core symptoms of schizotypy; however, he subsequently revised the role of ambivalence as a potentiating factor in schizotypy that increased the likelihood of developing schizophrenia [4]. Despite its prominence in Bleuler’s historical formulation of schizophrenia and Meehl’s model of schizotypy, the concept of ambivalence has subsequently received relatively little attention in the research and clinical literature regarding schizophrenia-spectrum psychopathology [5], and it is not part of the diagnostic formulation of any of the DSM-5-TR [6] schizophrenia spectrum or other psychotic disorders. The lack of inclusion likely resulted from a failure to adequately operationalize the construct, as well as its inclusion in psychoanalytic formulations of borderline personality (e.g., [7]).

The Schizotypal Ambivalence Scale (SAS; [8]) was developed to assess ambivalence relevant to schizotypy and the schizophrenia spectrum. Specifically, the 19 true–false items (see Table 1) emphasize the simultaneous experience of contradictory emotions or the rapid and almost random change of emotions over time. Sample items include, “My thoughts and feelings always seem to be contradictory”, and “Often I feel like I hate even my favorite activities”. The scale has good internal consistency (coefficient alpha = 0.84 in 1798 young adults) and test–retest (0.74 across nine weeks in 166 young adults) reliabilities [9].

Questionnaire, interview, and ambulatory assessment studies support the construct validity of the SAS. Multiple studies indicate that the SAS is associated with other measures of schizotypy (e.g., [9,10]). Kwapil et al. [11] reported that SAS scores correlated 0.49 with the positive schizotypy factor, 0.38 with the negative schizotypy factor, and 0.66 with the disorganized schizotypy factor of the Multidimensional Schizotypy Scale [12]. Burgin et al. [13] used experience sampling methodology to demonstrate that schizotypal ambivalence was associated with diminished positive affect, increased negative affect, as well as cognitive and social impairment in daily life. Using a French translation of the SAS [14], Loas et al. [15] found that first-degree relatives of people with schizophrenia had higher schizotypal ambivalence than first-degree relatives of people with non-psychotic psychiatric disorders. Finally, five interview studies with non-clinically ascertained young adults have provided converging evidence that the SAS is robustly associated with impaired functioning, schizophrenia-spectrum symptoms and personality disorder traits, and borderline personality disorder traits [9,11,16]. 

The empirical literature has supported the validity of the SAS. However, the scale was developed prior to the widespread availability of modern measurement tools such as item response theory (IRT) and differential item functioning (DIF), and the scale and its items have not undergone rigorous psychometric analysis. Furthermore, only one study, to our knowledge, has investigated the internal structure of the SAS. MacAulay et al. [10] examined the dimensional structure of the SAS in 334 participants. Using principal components analysis with an oblique rotation, they reported three underlying factors: interpersonal ambivalence, indecision/insecurity, and contradictory feelings. However, they used a Likert response format from 1 (*strongly disagree*) to 5 (*strongly agree*), and it is unclear if a multidimensional structure would be found using the original dichotomous scoring format.

### Goals and Hypotheses of the Present Study

Given the historical importance of ambivalence and the empirical promise demonstrated by the SAS, the present study provided a comprehensive assessment of the scale’s psychometric properties in a large sample of non-clinically ascertained young adults. The study examined the dimensionality of the SAS using the traditional dichotomous response option. Next, it employed classical test theory (CTT) and IRT metrics to examine key item properties (e.g., item difficulty, discrimination, and fit) and test properties (e.g., test information). Finally, we examined the possibility of item bias in the SAS with analyses of differential item functioning based on self-reported gender and racial-ethnic categories. We concluded with an overall evaluation of the scale’s psychometric features.

## 2. Method

### 2.1. Participants

The sample consisted of 7096 adults enrolled at the University of North Carolina at Greensboro (UNCG) who completed the scale as part of larger research projects or mass screening sessions. All participants provided informed consent, and the research was approved by the UNCG Institutional Review Board. The sample was predominantly female (5466 women, 1622 men) and young (*M* = 19.60 years, *SD* = 3.51, *Mdn* = 18.8, range = 18 to 64; age was available for approximately half of the sample). For self-reported racial and ethnic identification, the sample largely consisted of European American (*n* = 4861), African American (*n* = 1521), Asian (*n* = 199) Hispanic/Latinx (*n* = 148), Native American (*n* = 39), and people who endorsed other or no categories (*n* = 152).

### 2.2. Analytic Approach

The data were analyzed in R 4.2 [17] using the packages *psych* 2.2.3 [18], *TAM* 4.0.1 [19], and *lordif* 0.3.3 [20]. The IRT models were conducted using marginal maximum likelihood and were case centered for identification, yielding a trait theta score centered on zero. The raw data and R code are available at Open Science Framework (https://osf.io/ztycp/, accessed on 22 July 2021) for researchers who would like to duplicate the analyses and explore the data further.

## 3. Results

### 3.1. Descriptive Statistics, Reliability, and Dimensionality

Table 1 displays the descriptive statistics for each item, including the means (percent of participants who endorsed the binary item), standard deviations, and item-scale correlations, which ranged from 0.46 to 0.57. The scale exhibited good internal consistency reliability (Cronbach’s alpha = 0.84). Analysis of coefficient Omega (estimated with tetrachoric correlations) found that Omega-total was high (ω*_T_* = 0.93); omega-hierarchical, which reflects the item saturation of the general factor, was lower but nevertheless good (ω*_H_* = 0.71).

For dimensionality, we examined *essential unidimensionality*—a looser standard than strict unidimensionality that is commonly applied to psychological constructs [21]—using multiple criteria [22]. One of the more accepted methods is the ratio of the first and second eigenvalues, specifically a ratio of 4:1 [23] or 3:1 [21]. We also evaluated the minimum average partial (MAP) criterion and the scree plot from a parallel analysis [24]. The factor analyses were conducted in *psych* using maximum-likelihood factor analysis and tetrachoric correlations because of the dichotomous response scale. 

**Figure 1:**
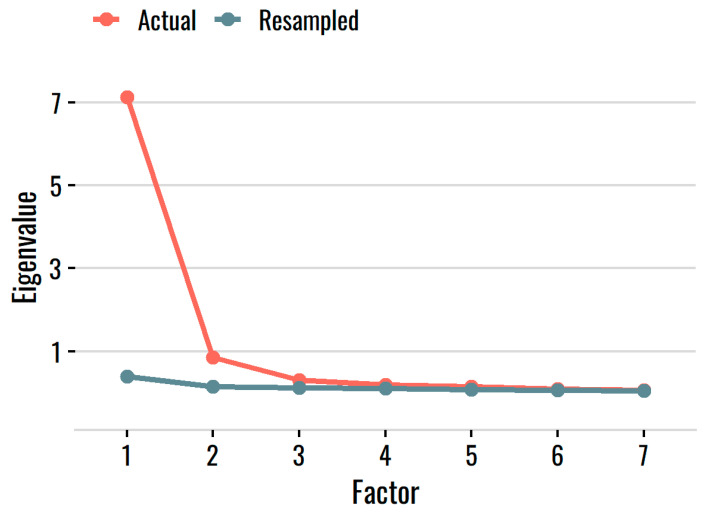
Actual and resampled eigenvalues from the parallel analysis. Note: Only the first 7 factors are shown for clarity.

The parallel analysis suggested seven factors based on the resampled values, but there was clearly one dominant factor and, at most, one minor secondary factor (see Figure 1). The MAP criterion suggested two factors, and the eigenvalue-ratio criterion suggested essential unidimensionality (a ratio of 8.4:1). To explore the small, secondary factor in more detail, we conducted an exploratory factor analysis with a bifactor rotation, which extracts a common, general factor and then specific orthogonal factors. All items had standardized loadings of at least 0.52–0.68 on the general factor, and no item had a larger loading on a specific factor than on the general factor. The specific factors appeared to reflect local dependence—pairs or small subsets of items that covaried with each other after accounting for the general factor—rather than substantive facets, a view that is supported by analyses of local independence in the next section. Taken together, the dominant first factor, high loadings of all items on the common factor, ratio of eigenvalues, and lack of substantive meaning of the specific minor factors suggest (1) good evidence for essential but not strict unidimensionality, and (2) that unidimensionality could probably be improved by trimming overlapping, partially redundant items that demonstrate local dependence.

### 3.2. IRT Model Fit and Local Independence

We estimated the IRT models in TAM using marginal maximum likelihood and case centering, which centers the trait theta score at 0. Two models were compared: a Rasch model that estimated item difficulty (*b*) parameters and a 2PL model that estimated both item discrimination (*a*) and difficulty (*b*) parameters. The models were compared using information theory metrics: the Akaike information criterion (AIC), Bayesian information criterion (BIC), and the Gilula–Haberman log penalty (GHP). All three criteria penalize model complexity to varying degrees and favor models with lower values. The 2PL model evidenced better fit than the Rasch model for the AIC (142,018 vs. 142,271), the BIC (142,279 vs. 142,408), and the GHP (0.527 vs. 0.528), suggesting the model fit improvement of the 2PL was sufficient to favor it despite including additional model parameters. We thus retained the 2PL model for the remaining analyses. Reliability for the estimated 2PL trait score was good (expected a posteriori reliability = 0.82).

The last step to evaluate the degree to which the 2PL model explained the data was to analyze whether the assumption of local independence holds. We evaluated the presence of locally dependent pairs using the adjusted *Q*_3_ statistic (a*Q*_3_), which corrects for bias in Yen’s [25] *Q*_3_ values by centering them on the average value [26]. Values of |0.20| (in the *r* metric) are common cut-offs for flagging locally dependent pairs [27]. No item pairs had a*Q*_3_ values greater than |0.20|, so local dependence was overall modest. We nevertheless explored the pairs with the highest a*Q*_3_ values to gain insight into the small, secondary factor found in the parallel analysis and the specific factors found in the exploratory bifactor analysis. The largest values were for items 14 and 16 (a*Q*_3_ = 0.18), items 11 and 15 (a*Q*_3_ = 0.15), and items 13 and 16 (a*Q*_3_ = 0.15); all remaining pairs were below 0.13. 

Although local dependence was overall low, with all a*Q*_3_ values being under |0.20|, the items with the highest values were the ones that formed specific factors in the bifactor analysis, and they have highly similar item wording or meaning that created relatively redundant pairs. In these cases, many of the items overlapped in using the word “love”, and they refer to ambivalence in the context of close, emotionally intimate relationships (see Table 1). A possible source of this local dependence is that the young adults in this sample likely interpret these items in terms of close romantic relationships (vs. parental or sibling relationships), so people with limited experience with such relationships (common among young adults high in negative schizotypy [28]) are less likely to endorse them. 

### 3.3. Item Characteristics

**Figure 2:**
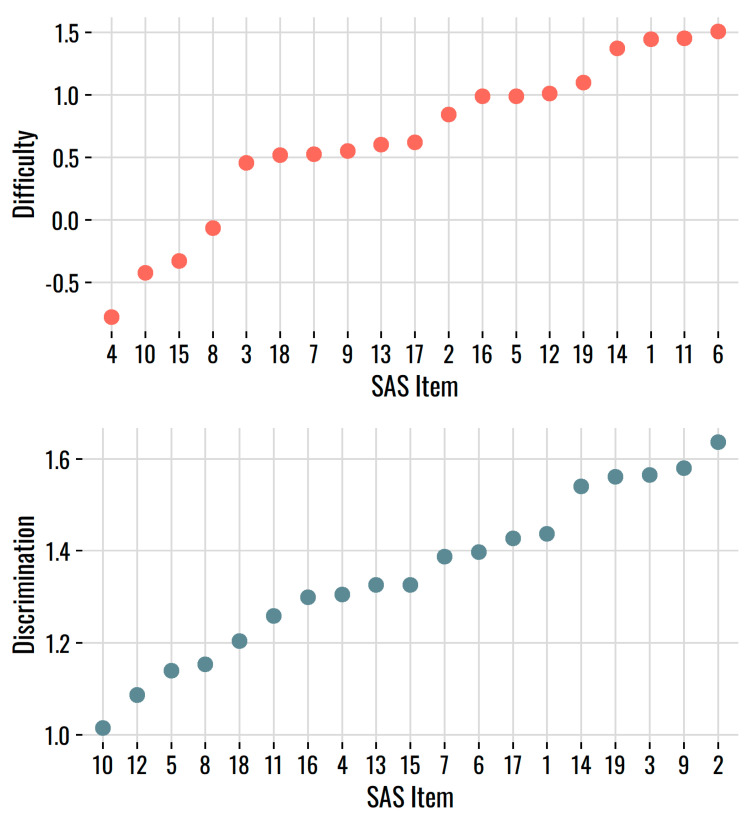
Item difficulty (*b*, **top panel**) and discrimination (*a*, **bottom panel**) values from the 2PL model, sorted from low to high.

The 2PL model provides estimates of the values for the items’ difficulty (*b*) and discrimination (*a*) parameters (see Table 1). The item difficulty values, reflecting the trait level at which someone has a 50:50 chance of endorsing the item, ranged from −0.78 to 1.51 (see Figure 2, top panel). Four items were relatively easy. Participants endorsed items 4, 8, 10, and 15 at a higher rate, and these four items had *b*-values less than 0. The remaining items had difficulty values roughly between 0.50 and 1.50, so most items were on the harder end of the scale, but no item had a *b* value greater than two logits.

The discrimination (*a*) parameters, shown in the bottom panel of Figure 2, showed good discrimination overall. The *a*-values ranged from 1.01 to 1.64, so even the lowest value is reasonable for a self-report measure of individual differences.

### 3.4. Test Information

**Figure 3:**
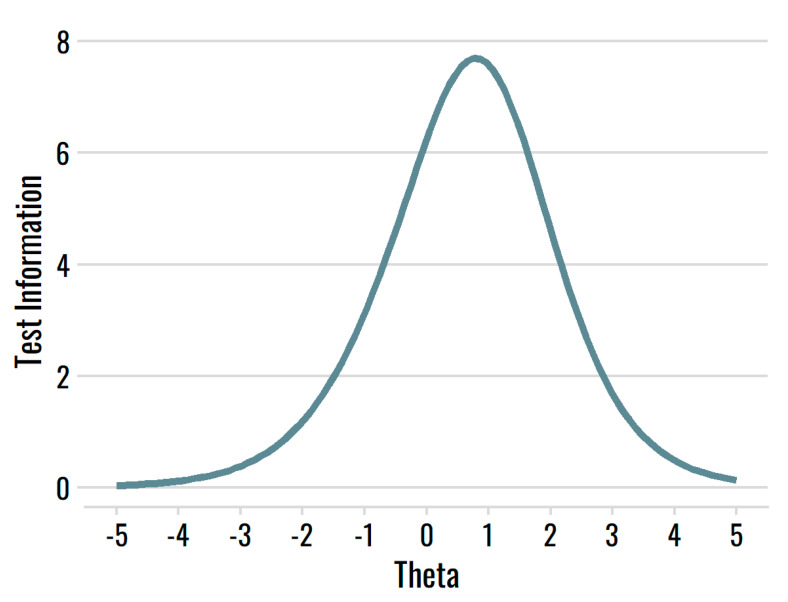
Test information function for the SAS.

Consistent with the profile of moderate item difficulty, the SAS had a test information function that peaked at *θ* = 0.78, the higher end of the trait scale (see Figure 3). Possessing more ambivalence indicates a higher risk for poor functioning, so it is thus apt for the scale to provide the most information at the higher end of the underlying trait.

### 3.5. Item Fit

**Figure 4:**
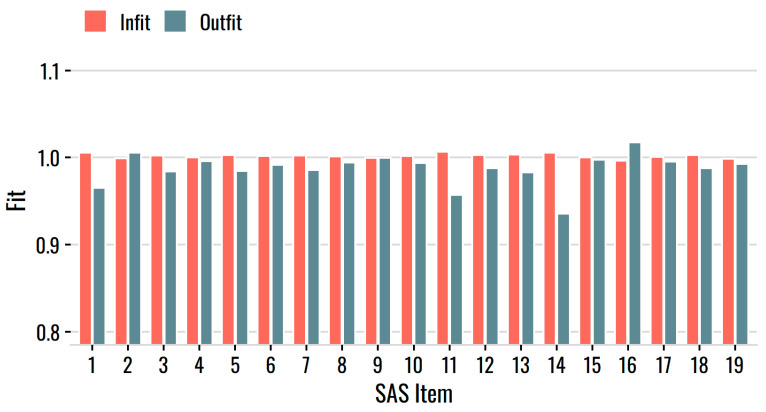
Infit and Outfit statistics for the SAS items.

To evaluate item fit, we considered Infit and Outfit, common item fit statistics based on mean-square residuals [29]. The expected value is 1, and values greater than 1 reflect underfitting items (i.e., noisier than expected). We applied a common threshold of 1.15 to flag underfitting items in this large sample. As Figure 4 shows, the values rarely exceeded 1, and never by very much.

**Figure 5:**
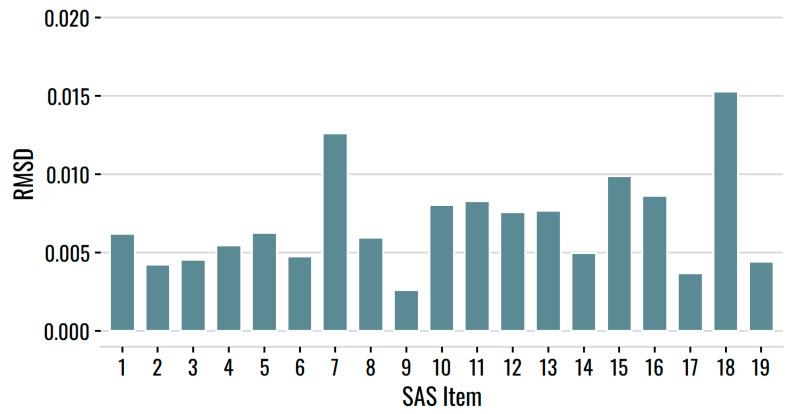
RMSD item fit for the SAS items.

A limitation of Infit and Outfit statistics, however, is that they can become insensitive in large samples [30]. We thus also evaluated item RMSD, a fit statistic that evaluates deviations between the true and fitted item response functions. In their work, Köhler et al. [31] suggested RMSD misfit benchmarks of *negligible* (RMSD < 0.02), *small* (0.02 ≤ RMSD < 0.05), *medium* (0.05 ≤ RMSD < 0.08), and *large* (RMSD ≥ 0.08). As Figure 5 illustrates, item fit was excellent for all 19 SAS items. RMSD values were below 0.02 for all items, suggesting that item misfit was negligible.

### 3.6. Differential Item Functioning

DIF was investigated to see if subgroups with the same true trait level varied in their likely responses. Such differences would reflect item bias—the operation of nuisance or construct-irrelevant factors—rather than true group differences in the underlying trait [32]. We used the logistic ordinal regression method implemented in *lordif* [33], which uses IRT-based trait scores and iterative purification methods to identify items showing uniform and non-uniform DIF [33,34]. A virtue of this approach is that it does not require specifying anchor items known to be DIF-free, which is important when relatively little is known about a scale’s item behavior. Because of our large sample size, we evaluated DIF via effect size statistics [35,36], particularly McFadden’s *R*^2^ [37], using a threshold of *R*^2^ = 0.02 (2% of the variance) to flag items for total DIF (i.e., uniform plus non-uniform DIF). Because *R*^2^ = 0.02 is a common benchmark for a “small effect size” in the *R*^2^ metric, it is a lenient threshold for an initial evaluation of items for possible DIF.

For gender, a comparison of women (*n* = 5466) and men (*n* = 1622) indicated that none of the 19 items was flagged for DIF using a *R*^2^ = 0.02 threshold, so any gender-based DIF is at most very small (see Table 1). To explore DIF further, we reduced the threshold to *R*^2^ = 0.01. Even at that level, no items were flagged were gender-based DIF. We thus conclude that the SAS items show essentially no gender-based DIF, inasmuch as an item bias effect less than *R*^2^ = 0.01 is too negligible to warrant attention.

For race and ethnicity, we recoded the categories into European-American participants (*n* = 4861) and participants of color (*n* = 1907). Participants in the declined-to-endorse/endorsed-another-category group (*n* = 152) were omitted for this analysis. Granted, this is a broad-brush approach that is limited by what was recorded in the original data. However, none of the 19 items was flagged for DIF using a *R*^2^ = 0.02 threshold, so DIF based on racial-ethnic identification is at most small for these items (see Table 1). To explore DIF further, we found that reducing the threshold to 0.01 yielded two items flagged for DIF: item 13 (“Everyone has a lot of hidden resentment toward his loved one”; *R*^2^ = 0.017) and item 16 (“I usually find that feelings of hate will interfere when I have grown to love someone”; *R*^2^ = 0.017). For both items, participants of color were more likely than European-American participants to endorse the item, given the same true trait level. There’s no apparent interpretation for these very small DIF effects, but it’s notable that these two items appeared among the handful of items with relatively larger local dependence.

## 4. Discussion

The SAS is one of the few self-report tools available for measuring ambivalence within the context of schizotypy and psychopathology more generally. We conducted a detailed psychometric evaluation of the SAS to discern its relative strengths and weaknesses and to examine lingering questions about dimensionality. Using a large sample of nearly 7100 adults, we applied IRT methods to examine the scale and item features and to determine whether items showed differential item functioning. We believe that the sample was appropriate for evaluating the SAS given that studies of schizotypy often focus on young adults who fall in an age range of greatest risk for developing schizophrenia-spectrum disorders. 

### 4.1. Dimensionality

Based on a group of criteria, the SAS appears to be essentially unidimensional. According to parallel analysis and MAP, there were at most two noteworthy factors, but the first was clearly dominant and greatly exceeded the 4:1 guidelines for eigenvalue ratios used for essential unidimensionality [21]. Subsequent exploratory bifactor analysis and local dependence statistics found evidence for a strong common factor and indicated that any minor factors were largely driven by locally dependent item pairs containing overlapping wordings or redundant meaning.

Our finding of essential unidimensionality conflicts with MacAuley et al. [10], who found a three-factor structure using exploratory procedures. However, one must consider the sample size, population, and item types. While the sample used in the present study included nearly 7100 respondents, the MacAuley et al. study included only 334 participants. While MacAuley et al. removed participants whose ages were outside of the range of 18–25 years, the current study did not exclude data based on participants’ ages, although the sample was predominantly within that age range. Finally, MacAuley et al. transformed the dichotomous SAS items into Likert-style items, which precludes a direct comparison of results, whereas our analyses used the original binary response scale and statistical models appropriate for such responses (e.g., parallel and factor analyses based on tetrachoric correlations). Taken together, given our much larger sample size and our administration of the scale in the format in which it was originally developed, we are inclined toward the evidence in favor of essential unidimensionality.

### 4.2. Item and Test Features

The SAS items fit a 2PL IRT model and showed limited evidence for item misfit. All items showed acceptable levels of item discrimination, so they all were effective at differentiating between people with different trait levels. Concerning item difficulty, on the whole, the items lean toward the “hard” end—it takes a relatively high level of the underlying schizotypal ambivalence trait to be likely to endorse them. The test information function is centered around 0.80, so the scale’s scores are most reliable in that region. This seems appropriate for a scale that is predominantly used in general samples, where there is more interest in reliably discriminating between participants on the higher end of the trait, and it follows from schizotypy scale development procedures in Kwapil et al. [12] and Gross et al. [38].

### 4.3. Differential Item Functioning

A notable finding was the lack of evidence for item bias. Analysis of differential item functioning found, at most, very small effect sizes for gender and for racial-ethnic identity. Given the large sample size, it’s reasonable to conclude that gender-based DIF is essentially zero for the SAS. This indicates that researchers can have confidence in any gender differences that are observed using the scale. Likewise, DIF based on racial and ethnic identification was at most very small. Only two items had *R*^2^ values greater than 1%, so DIF based on this categorization appears minimal. Because the underlying items are free of DIF, any group differences are likely to reflect true differences in the underlying trait, not specious differences driven by nuisance factors. Nevertheless, this issue deserves continued attention in future research, especially in light of the imbalanced group sizes and the relatively coarse classification of racial and ethnic identities that was afforded by the existing dataset.

### 4.4. Limitations and Conclusions

Several limitations of the present research should be noted. First, our sample consisted of adults who were not selected based on clinical features. The assessment of schizotypy has critical relevance for studies of at-risk and clinical samples (e.g., [12,39]), and the SAS has been used successfully in a small number of studies that recruited based on clinical criteria (e.g., psychiatric hospitalization or family history [14,15]) or that applied structured clinical interviews to assess a range of clinical disorders [9,11,16]. Nevertheless, it is not yet known how well the psychometric features of the SAS illustrated in the present sample would replicate in high-risk and clinical samples, and we believe that studying the scale’s performance in such samples is a major long-term goal for future research. Second, the present sample, while large, was nevertheless notably imbalanced regarding gender, and it was unable to provide fine differentiation for aspects of race and ethnicity. The large absolute numbers of men and women should suggest that the estimates are stable, but a key goal for future research should be to examine the psychometric qualities of the SAS in large samples that offer a more detailed look at the psychometric equivalence of the SAS across racial and ethnic identities. Finally, given the age of the SAS and subsequent advances in scale development, it is worth considering how these and future psychometric analyses could inform avenues for future revisions, such as rewording, adding, or omitting items.

The goal of the present study was to provide a comprehensive assessment of the psychometric properties of the SAS in a large sample of non-clinically ascertained young adults. Overall, the analyses support the psychometric features of the SAS: it appears to be essentially unidimensional, the items have an appropriate range of difficulty and good item discrimination, the items fit a 2PL model well, and DIF based on gender and racial and ethnic identity is at most minimal. The present psychometric findings, along with the growing empirical support from questionnaire, interview, and ambulatory assessment studies, support the continued use of the SAS. Furthermore, its relatively brief and non-invasive format makes it ideal for screening purposes and for inclusion in laboratory protocols. 

## Figures and Tables

**Table 1:** SAS items and descriptive statistics.

Item	Text	*Mean* (Percent Endorsed)	*SD*	Item-Scale Correlation (*r*)	IRT Difficulty	IRT Discrimination	Gender DIF (*R*^2^)	Race-Ethnicity DIF (*R*^2^)
1	Often I feel like I hate even my favorite activities.	0.18	0.38	0.47	1.45	1.44	0.000	0.002
2	My thoughts and feelings always seem to be contradictory.	0.28	0.45	0.56	0.84	1.64	0.000	0.001
3	My feelings about my own worth as a person are constantly changing back and forth.	0.38	0.49	0.57	0.45	1.57	0.000	0.006
4	Very often when I feel like doing something, at the same time I don’t feel like doing it.	0.68	0.46	0.49	−0.78	1.3	0.003	0.000
5	When I am trying to make a decision, it almost feels like I am physically switching from side to side.	0.29	0.45	0.48	0.99	1.14	0.000	0.000
6	It’s impossible to know how you feel because the people around you are constantly changing.	0.17	0.38	0.46	1.51	1.4	0.000	0.002
7	I always seem to be the most unsure of myself at the same time that I am most confident of myself.	0.37	0.48	0.54	0.52	1.39	0.001	0.004
8	I always seem to have difficulty deciding what I would like to do.	0.52	0.5	0.5	−0.07	1.15	0.001	0.004
9	Most people seem to know what they’re feeling more easily than I do.	0.36	0.48	0.57	0.55	1.58	0.000	0.000
10	Love and hate tend to go together.	0.59	0.49	0.47	−0.42	1.01	0.001	0.006
11	Love never seems to last very long.	0.19	0.4	0.46	1.45	1.26	0.001	0.007
12	The closer I get to people, the more I am annoyed by their faults.	0.29	0.45	0.47	1.01	1.09	0.001	0.000
13	Everyone has a lot of hidden resentment toward his loved one.	0.36	0.48	0.54	0.6	1.33	0.000	0.017
14	I have noticed that feelings of tenderness often turn into feelings of anger.	0.18	0.38	0.5	1.37	1.54	0.001	0.005
15	My experiences with love have always been muddled with great frustrations.	0.58	0.49	0.53	−0.33	1.33	0.000	0.000
16	I usually find that feelings of hate will interfere when I have grown to love someone.	0.27	0.45	0.51	0.99	1.3	0.000	0.017
17	A sense of shame has often interfered with my accepting words of praise from others.	0.35	0.48	0.55	0.62	1.43	0.000	0.002
18	I usually experience doubt when I have accomplished something that I have worked on for a long time.	0.38	0.49	0.51	0.52	1.2	0.000	0.002
19	I doubt if I can ever be sure exactly what my true interests are.	0.23	0.42	0.53	1.1	1.56	0.002	0.000

Note: *n* = 7096. For DIF, McFadden *R*^2^ values for total DIF are reported (e.g., a value of 0.017 is *R*^2^ = 0.017, or 1.7% of the variance).

## Data Availability

The raw data and R files used in the analyses are publicly available at the project’s Open Science Framework archive (https://osf.io/ztycp/, accessed on 22 July 2021).
